# Life-course body mass index trajectories and blood pressure in mid life in two British birth cohorts: stronger associations in the later-born generation

**DOI:** 10.1093/ije/dyv106

**Published:** 2015-06-13

**Authors:** Leah Li, Rebecca Hardy, Diana Kuh, Chris Power

**Affiliations:** ^1^Centre for Paediatric Epidemiology & Biostatistics and; ^2^MRC Unit for Lifelong Health and Ageing, University College London, UK

**Keywords:** Cohort study, blood pressure, BMI trajectories, joint modelling, life course

## Abstract

**Background:** Little is known about the impact of recent increases in obesity and more rapid gains in body mass index (BMI) on cardiovascular risk factors. We investigated life-course BMI trajectories associations with adult blood pressure (BP) across two generations.

**Methods:** We used the the 1946 and 1958 British birth cohorts. Joint multivariate response models were fitted to longitudinal BMI measures [7, 11, 16, 20, 26, 36, 43 and 50 y (years): 1946 cohort, *n* = 4787; 7, 11, 16, 23, 33 and 45 y: 1958 cohort, *n* = 16 820] and mid-adult BP. We adopted linear spline models with random coefficients to characterize childhood and adult BMI slopes.

**Results:** Mean systolic BP (SBP) decreased from the earlier- to later-born cohort by 2.8 mmHg in females, not males; mean diastolic BP (DBP) decreased by 3.2-3.3 mmHg (both sexes). Adult BMI was higher in the later- than the earlier-born cohort by 1.3-1.8 kg/m^2^, slopes of BMI trajectory were steeper from early adulthood and associations with adult BP were stronger. Associations between adult BMI and SBP were stronger in the later-born cohort. For males, childhood BMI slope was associated with SBP only in the later-born cohort; the association for adult BMI slope was stronger in the later-born cohort: correlation coefficient *r* = 0.28 [95% confidence interval (CI): 0.25,0.33] versus 0.13 (0.06,0.20). For females, childhood slope was associated with SBP in both cohorts; adult slope was associated with SBP only in the 1958 cohort [*r* = 0.34 (0.31,0.37)]. Patterns of child-to-adult BMI associations were similar in relation to DBP.

**Conclusions:** BP did not increase between two generations born 12 y apart despite higher BMI levels. A stronger association between BMI trajectory and BP in the later-born cohort suggests that BMI-related effects may have been offset by improvements in other factors linked to BP, such as diet and smoking.

Key Messages

- Mean values of systolic or diastolic blood pressure (BP) did not increase between two generations born 12 years apart, despite the steeper slope for BMI changes from early adulthood and higher levels of body mass index (BMI) in mid adulthood in the later- than the earlier-born cohort.
- A stronger association between life-course BMI trajectories and higher BP levels in the later-born cohort suggests that BMI-related effects may have been offset by improvements in other factors linked to BP over successive generations in the UK.

## Introduction

High blood pressure (BP) is a major risk factor for cardiovascular disease (CVD) in adults, contributing substantially to morbidity and mortality in the population.^1^^,^^2^ High body mass index (BMI) is associated with higher BP levels in children^3^ and adults.^4^ As many societies have become more obese during the recent epidemic, different generations have been affected at different life stages, such that some generations have had a steeper trajectory of BMI than others.^5^ It has been estimated that BMI has increased by 0.6 kg/m^2^ per decade in men and 0.4 kg/m^2^ in women in Western Europe during the past 30 years,^6^ and for every kg/m^2^ higher adult BMI, systolic BP (SBP) increases on average by approximately 1 mmHg.^4^ We would expect an increasing trend in population mean BP over the same period, by approximately 0.6 mmHg and 0.4 mmHg per decade respectively for men and women. However, mean adult BP levels and prevalence of hypertension have not increased (or even declined) during this period.^7^ This raises the prospect that the association of BMI (or life-course BMI trajectories) with adult BP may have weakened over successive generations. Such associations have been little studied but are of interest because of increasing levels of obesity across all ages. To study changes in associations between BMI trajectories and adult BP requires information on BMI across the life course and adult BP from different generations.

A comparison of the 1946 and 1958 British birth cohorts showed that changes in BMI trajectories had occurred, with the later-born cohort gaining BMI at a faster rate from early adulthood, and having a higher BMI by 1-2 kg/m^2^ in mid adulthood.^5^ The impact of the trends in BMI on BP levels is unclear. Separate studies of these cohorts show that high BMI and large BMI gain during adulthood were associated with higher adult BP levels,^8–10^ whereas childhood BMI was associated with adult BP only in the 1958 cohort. These studies^9^^,^^10^ used conditional regression models, which do not account for differences in the timing of measures between individuals or correlation of repeated measures within individuals. Such limitations can be overcome with joint multivariate response models. Given the lack of evidence on how the association between BMI trajectories and adult BP at an individual level has changed in recent decades, we used a joint multivariate response model for repeated BMI measures and adult BP for the 1946 and 1958 cohorts. Our aims were to investigate whether adult BP levels have changed between the two cohorts and whether the associations between characteristics of BMI trajectories (i.e. BMI levels or slope for BMI changes) and adult BP have changed.

## Methods

### Population

The 1946 birth cohort (MRC National Survey of Health and Development) includes children (*n* = 5362) from a socially stratified sample of single births to married women in 1 week in March 1946 in Britain. Cohort members were followed up from birth to age 60-64 y (*n* = 2661).^11^ For comparison purposes, our main analysis used data collected up to age 43 y (*n* = 3262). At age 43, contact was not attempted for individuals (*n* = 1882) who were living abroad (11.3% of original cohort), had died (6.8%) or had previously refused or could not be contacted (17%).^12^ The study received Multi-Centre Research Ethics Committee approval, and written informed consent was given by the participants.

The 1958 birth cohort includes all born in 1 week in March 1958 in Britain. Approximately 17 000 live births were followed up from birth to age 50 y. At age 45, 11 971 cohort members were invited to participate in a medical assessment by a trained nurse;^13^ 9377 participants provided information. Contact was not attempted for individuals (*n* = 5549) who were living abroad (7%), had died (6.7%) or had previously refused or could not be contacted (16.2%). In most respects the cohort followed to adulthood was representative of the original birth sample.^14^ Ethical approval for the 45-y data collection was given by the South East England Multi-Centre Research Ethics Committee. Our main analysis used data collected up to age 45 y.

### Measures

#### 

##### BMI measures

We used height and weight in the 1946 cohort measured at: 7, 11 and 15 y (to nearest in and lb); 36 and 43 y (0.5 cm and 0.5 kg); and 53 y (mm and 0.1 kg) in 1999 by trained personnel, and self-reported at ages 20 and 26 y. Height and weight in the 1958 cohort were measured at: 7, 11 and 16 y (in and lb); 33 y (cm and 0.1kg); and 45 y (mm and g) in 2003, and self-reported at age 23 y. Waist circumference (mm) was measured at 36, 43 and 53 y for the 1946 cohort, and at 45 y for the 1958 cohort. For females who were pregnant, self-reported pre-pregnancy weights were used (1946 cohort: *n* = 63 at age 26 y; 1958 cohort: *n* = 539 at 23 y), and for other ages measurements during pregnancy were excluded (1946 cohort: *n* = 107 at age 20 y, n = 30 at 36 y and *n* = 10 at 43 y; 1958 cohort: *n* = 229 at 33 y). BMI (kg/m^2^) at each age was calculated for both cohorts.

##### BP measures

For the 1946 cohort, BP was measured twice at age 43 (in 1989) with the participant seated for at least 5 min, using a Hawksley random zero sphygmomanometer (*n* = 3157). We used the average of two readings to compare with BP measures in the 1958 cohort at a similar age. Participants were asked whether they had taken any prescribed medication for high BP in the past year. For the 1958 cohort, BP was measured three times at age 45 with the participant seated for at least 5 min using an Omron automated device (*n* = 9297). We used the average of the first two readings. Participants were asked to show currently prescribed medications to the nurse, who coded antihypertensive drugs from direct observation of packaging.

### Statistical analysis

#### Adjustment for device, medication and age at examination

We made adjustments to standardize the BP outcome and improve comparability of measurements in the two cohorts. First, BP measures were taken with different devices: the Omron 705CP device tends to provide higher estimates than the sphygmomanometer.^15^ We converted BP measurements at age 45 y for the 1958 cohort to the equivalent sphygmomanometer readings, using previously derived formulae.^15^ Second, ignoring antihypertensive medication effects on BP measurements potentially leads to underestimation of the BMI/BP association;^16^ we adjusted BP measures by adding a constant of 10 mmHg to the observed SBP and diastolic BP (DBP) for those on treatment (1946 cohort: 3.4%; 1958 cohort: 4.6%).^16^^,^^17^ Third, levels of BP increase with age;^18^ we centred the 1946 cohort measures taken at age 43 to 45.2 y (mean age at measurement for the 1958 cohort) using the age trends estimated from growth models fitted to BP measures at 36, 43 and 53 y for the 1946 cohort. Waist circumference at 43 y for the 1946 cohort was also centred to 45.2 y using the age trends estimated from measures at 36, 43 and 53 y.

#### Joint modelling for BMI trajectories and adult BMI

**Figure 1.**
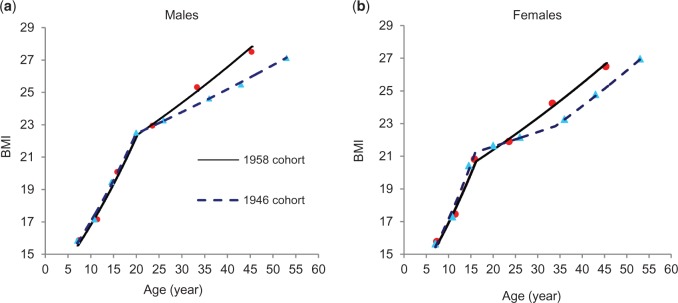
Observed (dots) and estimated (lines) (estimated using joint multivariate models) geometric mean BMI in the 1946 and 1958 cohorts.

From age 7 y, there was a maximum of eight BMI measures per person in the 1946 cohort and six in the 1958 cohort. To establish how longitudinal changes of BMI during different periods were associated with BP in mid adulthood, we applied a joint modelling approach to three response variables: repeated BMI measures (log10 transformed to correct for the right skewness) and SBP and DBP levels at 45 y. The observed (geometric) mean BMI had distinct trajectories in childhood and adulthood and differed by sex (Figure 1a, b). We adopted a linear spline function to summarize the longitudinal changes of BMI. For the 1958 (both sexes) and 1946 (males) cohorts, we used one knot ($t_{0}$) to allow for distinct BMI curves in ‘childhood’ and ‘adulthood’. For adult SBP and DBP, we applied single-level models and specified only an individual-specific (random) intercept ($\beta_{3j}$ and $\beta_{4j}$) to represent BP values for individual *j*, as each individual was measured once. The joint model is written as:

(i) $$\begin{array}{c} \text{log}BMI_{ij}=\beta_{0j}+\beta_{1j}t_{ij}+\beta_{2j}(t_{ij}-t_{0})I_{t_{ij}>t_{0}}+e_{ij}\\ SBP_{j}=\beta_{3j}\\ DBP_{j}=\beta_{4j} \end{array}$$

where $t_{ij}$ is the exact age at measurement *i*, $I_{t_{ij}>t_{0}}$ represents two age ranges. For $\text{log }BMI_{ij}$ there are individual-specific childhood slope $\beta_{1j}$ (for $t_{ij}\leq t_{0}$) and adult slope $\beta_{1j}+\beta_{2j}$ (for $t_{ij}>t_{0}$). For females in the 1946 cohort, the slope increased after mid-30 ages (Figure 1b). We included an additional knot ($t_{1}$) in the model for log BMI:

(ii) $$\begin{array}{c} \begin{array}{c} \text{log}BMI_{ij}=\beta_{0j}+\beta_{1j}t_{ij}+\beta_{2j}(t_{ij}-t_{0})I_{t_{ij}>t_{0}}\\ +\beta_{2"j}(t_{ij}-t_{1})I_{t_{ij}>t_{1}}+e_{ij} \end{array}\\ SBP_{j}=\beta_{3j}\\ DBP_{j}=\beta_{4j} \end{array}$$

Models i and ii were fitted in MLwiN. The knots were determined based on the likelihood profile: $t_{0}$ was chosen at 20 y for males and 16 y for females. The individual characteristics include BMI level at any age (t) and slopes for $\text{log }BMI_{ij}$ in childhood (*t* ≤ 20 y for males; *t* ≤ 16 y for females) and in adulthood (*t > *20 y and *t* > 16 y, respectively). For females of the 1946 cohort, the knot $t_{1}$ was chosen at 34 y. Their BMI trajectories were characterized by three linear curves, with slopes for childhood (*t* ≤ 16 y), early adulthood (16 y < *t* < 34 y) and mid adulthood (*t* ≥ 34 y). The associations (i.e. correlations coefficients) between characteristics of BMI trajectories and adult BP and 95% confidence intervals (CI) were estimated using the nonparametric bootstrap re-sampling procedure. We also conducted additional analyses to establish whether our findings were consistent based on estimated regression coefficients of BP on BMI slopes, using covariance matrix. Details of model assumptions and estimation are provided in supplementary material, available as Supplementary data at *IJE* online.

Numbers of participants with BMI measures decreased with age in both cohorts due to loss to follow-up. However, the joint models were applied to participants with at least one measure of BMI and/or BP and thus most were included: total 21 607 individuals (4787 in the 1946 cohort, 16820 in the 1958 cohort). Of these, 12 487 (3157 and 9330, respectively) had adult BP measures. We repeated analysis using only individuals with adult BP measures to assess whether the BMI trajectories for those with a BP measurement differed from all cohort members.

## Results

In females, adult SBP decreased on average by 2.8 mmHg (95% CI: 1.9,3.7) between the 1946 and 1958 cohorts but no difference was observed for males; whereas, mean DBP decreased by 3.3 mmHg (2.7,3.9) in males and 3.2 mmHg (2.6,3.8) in females (Table 1). The slope of BMI increase from early adulthood was significantly steeper in the 1958 cohort compared with the 1946 cohort. Thus BMI trajectories diverged during adulthood. For males, the slope (for log *BMI*) was 0.0038 (equivalent to 0.9% increase in BMI per year) in the 1958 cohort and 0.0025 (0.6% per year) in the 1946 cohort. For females, the respective slopes were 0.0038 and 0.0018 (0.9% and 0.4% per year) until the mid 30s, and were similar thereafter (0.9% per year) (Table 1).

**Table 1.** Characteristics of SBP, DBP and BMI (mean, 95% CI) for the 1946 and 1958 cohorts estimates using joint multivariate models

	1946 cohort (*n* = 4787)		1958 cohort (*n* = 16 820)	
Males	Mean	95% CI	Mean	95% CI
SBP at 45 y (mmHg)	126.8	(126.1, 127.6)	126.8	(126.4, 127.2)
DBP at 45 y (mmHg)*	83.8	(83.2, 84.4)	80.5	(80.3, 80.8)
Child slope^a^ (7_20 y)	0.0120	(0.0118, 0.0122)	0.0121	(0.0120, 0.0122)
Adult slope^a^ ( ≥ 20 y)*	0.0025	(0.0024, 0.0026)	0.0038	(0.0037, 0.0039)
Waist at 45 y (cm)*	93.2	(92.7, 93.7)	98.4	(98.1, 98.7)
**Females**				
SBP at 45 y (mmHg)	123.5	(122.6, 124.3)	120.7	(120.3, 121.0)
DBP at 45 y (mmHg)	79.3	(78.7, 79.9)	76.1	(75.9, 76.4)
Child slope^a^ (7-16y)*	0.0154	(0.0151, 0.0156)	0.0142	(0.0140, 0.0143)
Adult slope^a^ (16-34 y)*	0.0018	(0.0016, 0.0019)	0.0038	(0.0037, 0.0039)
Adult slope^a^^ ^≥ 34 y	0.0038	(0.0036, 0.0039)		
Waist at 45 y (cm)*	79.7	(79.2, 80.3)	85.5	(85.1, 85.9)

^a^Slope for logBMI.

*P < 0.05 for the test of difference in characteristics of BP and BMI between cohorts.

### Associations between characteristics of BMI trajectories and adult BP

**Figure 2.**
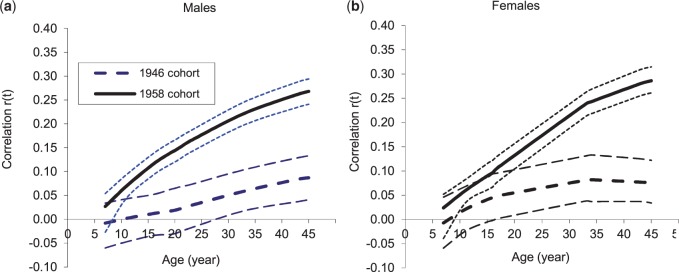
Correlation coefficients (95% CI) between log *BMI* and SBP at 45 y in the 1946 and 1958 cohorts estimated using joint multivariate models.

**Figure 3.**
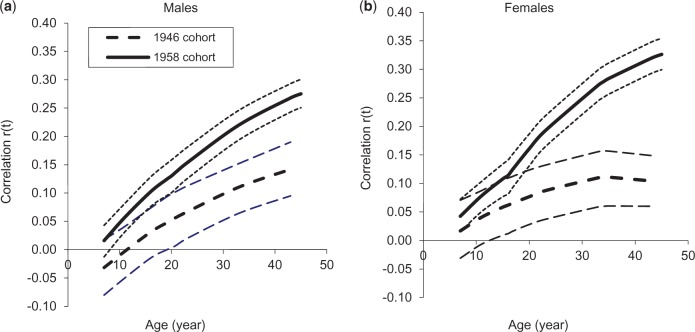
Correlation coefficients (95% CI) between log *BMI* and DBP at 45 y in the 1946 and 1958 cohorts estimated using joint multivariate models.

The correlations between BMI from ages 7 y to 45 y and adult SBP and DBP are illustrated in Figures 2 and 3. BMI at 7 y was not correlated with adult BP, except with DBP among females in the 1958 cohort (Table 2). Thereafter, for both sexes, cohorts and for SBP and DBP, associations strengthened with increasing age of BMI measurement, although more rapidly in the 1958 cohort than the 1946 cohort. For SBP, the association with BMI was significant by 11 y in the 1958 cohort (both sexes) and by early adulthood in the 1946 cohort (late 20s for males and late teens for females) (Figure 2). The correlation between BMI at 45 y and SBP was significantly greater in the later- than the earlier-born cohort: *r* = 0.27 (0.24,0.29) versus 0.09 (0.04,0.13) for males and 0.29 (0.26,0.32) versus 0.08 (0.03,0.12) for females. The correlation between waist circumference and SBP was also greater in the later- than the earlier-born cohort: 0.24 (0.22,0.27) versus 0.12 (0.07,0.17) for males; 0.30 (0.27,0.32) versus 0.13 (0.08,0.18) for females (Table 2).

**Table 2.** Correlation coefficients (95% CI) between BMI characteristics and SBP and DBP at age 45 y for the 1946 and 1958 birth cohorts estimated using joint multivariate models

	Systolic blood pressure (SBP) at 45 y				Diastolic blood pressure (DBP) at 45 y			
	1946 cohort (*n* = 4787)		1958 cohort (*n* = 16 820)		1946 cohort (*n* = 4787)		1958 cohort (*n* = 16 820)	
Males	r	95% CI	r	95% CI	r	95% CI	r	95% CI
BMI7	−0.008	(−0.060, 0.034)	0.027	(−0.027, 0.054)	−0.029	(−0.078, 0.021)	0.025	(−0.002, 0.051)
BMI11	0.002	(−0.047, 0.043)	0.070	(0.045, 0.094)	−0.003	(−0.047, 0.042)	0.064	(0.038, 0.089)
BMI16	0.012	(−0.034, 0.052)	0.116	(0.093, 0.138)	0.036	(−0.011, 0.077)	0.106	(0.082, 0.129)
BMI20	0.019	(−0.030, 0.065)	0.144	(0.120, 0.166)	0.057	(0.006, 0.102)	0.132	(0.108, 0.153)
BMI23	0.029	(−0.018, 0.073)	0.165	(0.141, 0.186)	0.071	(0.023, 0.116)	0.156	(0.133, 0.178)
BMI26	0.039	(−0.009, 0.084)	0.183	(0.161, 0.206)	0.085	(0.039, 0.128)	0.180	(0.156, 0.202)
BMI33	0.059	(0.018, 0.104)	0.222	(0.198, 0.246)	0.113	(0.069, 0.156)	0.227	(0.204, 0.251)
BMI43	0.084	(0.037, 0.129)	0.262	(0.235, 0.288)	0.145	(0.101, 0.186)	0.277	(0.253, 0.302)
BMI45	0.087	(0.041, 0.133)	0.268	(0.241, 0.294)	0.150	(0.105, 0.191)	0.285	(0.260, 0.311)
Child slope^a^ (7-20 y)	0.044	(−0.028, 0.122)	0.208	(0.170, 0.241)	0.130	(0.049, 0.201)	0.188	(0.166, 0.209)
Adult slope ^a^ (>20 y)	0.125	(0.061, 0.196)	0.283	(0.245, 0.327)	0.181	(0.112, 0.240)	0.337	(0.299, 0.379)
Waist circumference at 45 y	0.119	(0.070, 0.168)	0.244	(0.216, 0.273)	0.167	(0.115, 0.213)	0.264	(0.237, 0.291)
**Females**								
BMI7	−0.007	(−0.059, 0.046)	0.024	(−0.039, 0.052)	0.022	(−0.031, 0.069)	0.043	(0.016, 0.074)
BMI11	0.021	(−0.024, 0.068)	0.060	(0.034, 0.082)	0.044	(0.000, 0.089)	0.079	(0.052, 0.105)
BMI16	0.045	(−0.002, 0.094)	0.096	(0.067, 0.122)	0.065	(0.018, 0.112)	0.116	(0.083, 0.143)
BMI20	0.056	(0.013, 0.103)	0.138	(0.110, 0.162)	0.081	(0.041, 0.127)	0.162	(0.132, 0.188)
BMI23	0.063	(0.022, 0.109)	0.166	(0.139, 0.190)	0.092	(0.054, 0.135)	0.193	(0.165, 0.219)
BMI26	0.069	(0.027, 0.116)	0.191	(0.164, 0.215)	0.102	(0.062, 0.148)	0.221	(0.194, 0.248)
BMI33	0.081	(0.038, 0.131)	0.238	(0.213, 0.262)	0.119	(0.070, 0.167)	0.273	(0.246, 0.300)
BMI43	0.077	(0.037, 0.125)	0.280	(0.255, 0.309)	0.114	(0.675, 0.158)	0.332	(0.303, 0.362)
BMI45	0.076	(0.034, 0.122)	0.286	(0.261, 0.315)	0.112	(0.065, 0.156)	0.327	(0.300, 0.354)
Child slope^a^ (7-16 y)	0.107	(0.016, 0.211)	0.185	(0.115, 0.258)	0.110	(0.014, 0.205)	0.191	(0.119, 0.256)
Adult slope^a^ (16-34 y)	0.066	(−0.008, 0.140)	0.339	(0.307, 0.372)	0.098	(0.023, 0.171)	0.379	(0.345, 0.412)
Adult slope^a^ (> 34 y)	0.007	(−0.082, 0.088)			0.009	(−0.070, 0.088)		
Waist circumference at 45 y	0.127	(0.078, 0.176)	0.295	(0.267, 0.322)	0.164	(0.115, 0.213)	0.330	(0.303, 0.357)

^a^Slope for logBMI

Rate of BMI change was positively associated with adult BP and the strength of the association differed between cohorts. For males, childhood slope for BMI growth was associated with SBP in the 1958 cohort [*r* = 0.21 (0.17,0.24)] but not in the 1946 cohort. Adult slope for BMI increase was associated with SBP, and the association was stronger in the later [*r* = 0.28 (0.25,0.33)] than the earlier-born cohort [*r* = 0.13 (0.06,0.20)]. For females, childhood slope was associated with SBP in both cohorts: *r* = 0.11 for the 1946 cohort and 0.19 for the 1958 cohort. The slope for BMI increase in adulthood (16-45 y) was positively associated with SBP in the 1958 cohort (*r* = 0.34), whereas in the 1946 cohort, there was no association with the BMI slope (16-34 y) and thereafter (Table 2). For DBP, the patterns of association with child-to-adult BMI were similar to those for SBP, except the association with BMI was significant from an earlier age in the 1946 cohort (Table 2, Figure 3). As the BMI/BP association strengthened between cohorts, mean BP increased more rapidly with increasing BMI in the later-born cohort. Therefore the difference in mean adult BP (negative, except for SBP of males) levelled off with increasing BMI. In addition, the estimated characteristics for BMI trajectories and their associations with adult BP were little affected by restriction to individuals with BP measures (data not shown). The results based on regression coefficients for BP on BMI slopes are broadly similar to those presented for correlation coefficients (Supplementary Table S1, available as Supplementary data at *IJE* online).

## Discussion

Between the 1946 and 1958 cohorts, average SBP (females) and DBP (both sexes) declined by ≈ 3 mmHg, whereas SBP in males was unchanged. Yet, the slope for BMI changes was steeper from early adulthood and adult BMI was higher in the later- than the earlier-born cohort by 1.3-1.8 kg/m^2^. In general, a steeper BMI slope in childhood and adulthood was associated with increased BP, more consistently and strongly in the later-born generation. Specifically, associations with adult BP were evident for BMI at an earlier age in the later- than the earlier-born generation and the strengthening associations for BMI with increasing age were more rapid. Thus, the associations of adult BMI and waist circumference with BP were stronger in the 1958 cohort (*r* = 0.24-0.30 versus 0.09-0.13). Our findings suggest that associations of BMI trajectories with adult BP have changed over the 12-year interval.

### Methodological considerations

Exploring life-course BMI trajectories’ associations with adult BP is methodologically challenging.^8^ Previous analyses of the same cohorts used conditional regression^9^^,^^10^ treating repeated BMI measures as independent variables, expressed in terms of the baseline measure and subsequent increments.^19^ The resulting association with BP of BMI increases cannot be directly compared, as timing of BMI measures differed across cohorts. Other studies have used a two-step approach^20–22^ where individual growth characteristics estimated from a random effect model (thus subject to error) are used as independent variables in the primary model for an outcome.

The joint multivariate response model applied here has several major strengths: (i) it directly relates individual characteristics of BMI trajectories to BP through the covariance of the model coefficients, thus accounting for within-individual correlations between repeated BMI measures and between BMI and BP; and (ii) it includes individuals with different numbers and timing of BMI measures, or with incomplete data (our analysis included > 90% of the original cohorts). Linear spline models with random coefficients for repeated BMI measurements allow estimation of the associations (indicated by correlations) of BMI at any age, or distinct childhood and adulthood BMI slopes, with adult BP.^8^

The two population cohorts have many common characteristics and also some differences. Ages of contact were not always directly comparable, but we used information collected at additional time-points to derive estimates for BP for comparable ages. Since BP measured with different devices could introduce bias, hence, we standardized measurements to the mercury sphygmomanometer.^15^ BP measurements would be affected by BP-lowering medication, so adjustments were applied for those on treatment.^16^ Furthermore, findings did not differ from those presented when excluding individuals on medication, or when restricting analyses to individuals with BP measures.

### Comparison with other studies

Studies of adult BP trends in the UK and elsewhere in Western Europe show a decline in the last 20-30 y.^6^^,^^7^^,^^23^ We found an estimated decline of 0.25 mmHg/year in SBP for females (1989-2003) but no decline for males. In England, adult SBP was reported to have reduced more in women (0.5 mmHg/year) than in men (0.2 mmHg/year) (1994-2003).^24^ DBP declined by 0.25 mmHg/year among both sexes in our study. It has been shown that mean population DBP fell by 7.7% in England and Wales (1981-2000).^25^ A cross-sectional association of high BMI with elevated BP is well established in children and adults.^18^ Few studies have explored the impact of BMI or BMI changes across life on adult BP. A strong positive association between changes of adult weight^26^ and BP was found in separate analysis of the 1946 and 1958 cohorts,^9^^,^^27^ and BMI gains in childhood were associated with SBP in the 1958 cohort but only in females in the 1946 cohort.^9^^,^^10^ Published evidence is scarce on how the BMI/BP association has changed over time with rising obesity prevalence. Interestingly, a weakened BMI/BP association has been reported in African (1989-2004)^28^ and Chinese (1996-2006)^29^ populations: while mean BMI increased, mean BP declined^28^ or changed little.^29^ With opposite trends in BP and BMI, we might also expect a weakening of the association across cohorts. However, our results indicate that the association of adult BP with BMI started at an earlier age, and slopes for BMI increases both in childhood and adulthood have a stronger association with BP in the later-born cohort.

### Potential explanations

Although the cohort-specific slopes for increasing BMI with age examined here cover a substantial period of life (7-45 y), they may be too small in magnitude to affect BP levels at 45 y. For example, the increase in adult slope (for log *BMI*) for men between the two cohorts was only 0.0013 ( = 0.0038 - 0.0025). We would expect an increase in slope of this magnitude to be associated with an increase in mean SBP of 0.7 mmHg (for a correlation between adult slope and BP of 0.125 in the 1946 cohort) or 1.5 mmHg (0.283 in the 1958 cohort). Thus the magnitude of the difference in slope is sufficient to lead to a substantial increase in mean BP. Slopes of this magnitude are important determinants for BP and the magnitude of slopes has increased between the two cohorts from early adulthood. Yet we found that mean BP levels were lower or not different in the later- versus the earlier-born cohort, opposing the BMI trends, implying that other determinants for adult BP (possibly other CVD risk factors) may have changed over time.

The strengthening BMI/BP association could reflect changes in the meaning of BMI. BMI does not differentiate between fat and lean mass which have opposing effects on CVD risk. A high BMI in the earlier-born cohort might indicate greater muscle mass than in the later-born cohort, for whom it might represent greater fat mass. The faster tempo of childhood growth and weight gain, more rapid increases in adult BMI and larger waist of the later-born cohort^5^ potentially support the possibility that they had more fat mass than the earlier-born cohort. There is also the possibility that differential sample attrition has affected the cohort comparison. Levels of and explanations for sample loss are broadly similar:^30^^,^^31^ the mortality rate after birth until mid adulthood was almost identical (1946 cohort: 6.8%; 1958 cohort: 6.7%) and infant mortality fell from 3.5% (1946 cohort) to 2% (1958 cohort). Therefore the slightly higher mortality rate after infancy, or possibility of higher non-response among those with elevated BP in the 1958 than in the 1946 cohort, may partly explain the lack of increase in BP levels between cohorts. However, it is unclear how such differences could account for the strengthening BMI/BP association over time.

Mean adult height, known to be inversely associated with BP, has increased between cohorts,^5^ but the strengthening BMI/BP association is not accounted for by trends in height (data not shown). The greater height (particularly greater leg length), which reflects better early life environment,^32^ may partly explain the lower BP in in the 1958 cohort. Low birthweight is associated with elevated adult BP.^33^ Birthweights < 2.5k g were slightly more prevalent in the later- (4.8%) than earlier-born cohort, although were low overall (4.3%) and mean birthweight differed little.^5^ Improvement in other risk factors could have contributed to the decline of BP.^34^ Smoking is a putative risk factor for elevated BP,^35^ although evidence is inconsistent,^36^ and smoking is also associated with low BMI. In England and Wales, mean BP declined when smoking prevalence fell by 35% between 1981 and 2000.^25^ The decline in smoking from 30.9% (men) and 29.0% (women) in the 1946 cohort to 25.1% and 23.3%, respectively, in the 1958 cohort (unpublished observation) might explain lower BP levels in the later-born. Reduced saturated fat and increased fruit and vegetable consumption may have a beneficial contribution to BP in the population,^37^ but they would also be associated with lower BMI. Changes in other possible factors linked to raised BP, such as high salt intake and lowered physical activity levels, may also be affecting trends in BP over the period studied here.

Early detection and treatment for high BP has contributed to the reduction in coronary heart disease in England and Wales.^38^ We have made adjustment to BP measurements for those on medication, but the treatment effect may have improved over time.^39^ However, this is unlikely to have a substantial impact on the BP trends given the small proportion on medication at ages 43-45 y. A recent study shows that BP treatment had only a modest effect on declines in BP in English adults.^40^

To conclude, a strengthening association between life-course BMI gains and BP between two generations of contemporary adults, but with no detrimental trend in BP, suggests that BMI-related effects have been offset by improvements in other factors linked to BP, such as diet and smoking. Further studies should investigate how risk factors for high BP may have changed over time, and the implications of the strengthening BMI/BP association for subsequent cohorts with increasing levels of obesity.

## Supplementary Data

Supplementary data are available at *IJE* online.

## Funding

LL was funded by the Medical Research Council (MRC) Career Development Award in Biostatistics (grant G0601941). This work was undertaken at GOSH/UCL Institute of Child Health which received a proportion of funding from the Department of Health's NIHR Biomedical Research Centres funding scheme. The Centre for Paediatric Epidemiology and Biostatistics benefited from funding support provided by the MRC in its capacity as the MRC Centre of Epidemiology for Child Health (grant G0400546). The MRC has funded the 1946 cohort since 1962 (grants MC_UU_12019/2 and MC_UU_12019/1), and also the 45-year survey of the 1958 cohort (grant G0000934).

## Supplementary Material

Supplementary Data

## References

[1] Lewington S Clarke R Qizilbash N Peto R Collins R Age-specific relevance of usual blood pressure to vascular mortality: a meta-analysis of individual data for one million adults in 61 prospective studies. Lancet 2002;360**:** 1903–13. 12493255 10.1016/s0140-6736(02)11911-8

[2] Ezzati M Lopez AD Rodgers A Vander HS Murray CJ Selected major risk factors and global and regional burden of disease. Lancet 2002;360**:** 1347–60. 12423980 10.1016/S0140-6736(02)11403-6

[3] Schiel R Beltschikow W Kramer G Stein G Overweight, obesity and elevated blood pressure in children and adolescents. Eur J Med Res 2006;11**:** 97–101. 16751109

[4] Whitlock G Lewington S Sherliker P Body-mass index and cause-specific mortality in 900 000 adults: collaborative analyses of 57 prospective studies. Lancet 2009;373**:** 1083–96. 19299006 10.1016/S0140-6736(09)60318-4 PMC2662372

[5] Li L Hardy R Kuh D Lo CR Power C Child-to-adult body mass index and height trajectories: a comparison of 2 British birth cohorts. Am J Epidemiol 2008;168**:** 1008–15. 18801885 10.1093/aje/kwn227 PMC3159394

[6] Finucane MM Stevens GA Cowan MJ National, regional, and global trends in body-mass index since 1980: systematic analysis of health examination surveys and epidemiological studies with 960 country-years and 9.1 million participants. Lancet 2011;377**:** 557–67. 21295846 10.1016/S0140-6736(10)62037-5 PMC4472365

[7] Danaei G Finucane MM Lin JK National, regional, and global trends in systolic blood pressure since 1980: systematic analysis of health examination surveys and epidemiological studies with 786 country-years and 5.4 million participants. Lancet 2011;377**:** 568–77. 21295844 10.1016/S0140-6736(10)62036-3

[8] Li L Child-to-Adult BMI Trajectories and Cardiovascular Disease Risk Factors in Mid-Life: A Joint Multivariate Modeling Approach. Miami, FL: American Statistical Association, 2011.

[9] Li L Law C Power C Body mass index throughout the life-course and blood pressure in mid-adult life: a birth cohort study. J Hypertens 2007;25**:** 1215–23. 17563534 10.1097/HJH.0b013e3280f3c01a

[10] Hardy R Wadsworth ME Langenberg C Kuh D Birthweight, childhood growth and blood pressure at 43 years in a British birth cohort. Int J Epidemiol 2004;33**:** 121–29. 15075157 10.1093/ije/dyh027

[11] Kuh D Pierce M Adams J Cohort profile: updating the cohort profile for the MRC National Survey of Health and Development: a new clinic-based data collection for ageing research. Int J Epidemiol 2011;40**:** e1–e9. 21345808 10.1093/ije/dyq231 PMC3043283

[12] Wadsworth M Kuh D Richards M Hardy R Cohort Profile: The 1946 National Birth Cohort (MRC National Survey of Health and Development). Int J Epidemiol 2006;35**:** 49–54. 16204333 10.1093/ije/dyi201

[13] Power C Elliott J Cohort Profile: The 1958 British birth cohort (National Child Development Study). Int J Epidemiol 2006;35**:** 34–41. 16155052 10.1093/ije/dyi183

[14] Ferri E Life at 33:the Fifth Follow-Up of the National Child Development Study. London: National Children's Bureau, 1993.

[15] Stang A Moebus S Mohlenkamp S Algorithms for converting random-zero to automated oscillometric blood pressure values, and vice versa. Am J Epidemiol 2006;164**:** 85–94. 16675536 10.1093/aje/kwj160

[16] Tobin MD Sheehan NA Scurrah KJ Burton PR Adjusting for treatment effects in studies of quantitative traits: antihypertensive therapy and systolic blood pressure. Stat Med 2005;24**:** 2911–35. 16152135 10.1002/sim.2165

[17] Law MR Wald NJ Morris JK Jordan RE Value of low dose combination treatment with blood pressure lowering drugs: analysis of 354 randomised trials. BMJ 2003;326**:** 1427. 12829555 10.1136/bmj.326.7404.1427 PMC162261

[18] Wills AK Lawlor DA Matthews FE Life course trajectories of systolic blood pressure using longitudinal data from eight UK cohorts. PLoS Med 2011;8**:** e1000440. 21695075 10.1371/journal.pmed.1000440 PMC3114857

[19] De Stavola BL Nitsch D dos Santos Silva I Statistical issues in life course epidemiology. Am J Epidemiol 2006;163:84–96. 16306313 10.1093/aje/kwj003

[20] Ben-Shlomo Y McCarthy A Hughes R Tilling K Davies D Davey Smith G Immediate postnatal growth is associated with blood pressure in young adulthood: the Barry Caerphilly Growth Study. Hypertension 2008;52**:** 638–44. 18768401 10.1161/HYPERTENSIONAHA.108.114256

[21] Fraser A Hughes R McCarthy A Early life growth and hemostatic factors: the Barry Caerphilly Growth study. Am J Epidemiol 2008;168**:** 179–87. 18495627 10.1093/aje/kwn106

[22] Tzoulaki I Sovio U Pillas D Relation of immediate postnatal growth with obesity and related metabolic risk factors in adulthood: the northern Finland birth cohort 1966 study. Am J Epidemiol 2010;171**:** 989–98. 20360243 10.1093/aje/kwq027

[23] Kastarinen MJ Nissinen AM Vartiainen EA Blood pressure levels and obesity trends in hypertensive and normotensive Finnish population from 1982 to 1997. J Hypertens 2000;18**:** 255–62. 10726710 10.1097/00004872-200018030-00003

[24] Primatesta P Poulter NR Improvement in hypertension management in England: results from the Health Survey for England 2003. J Hypertens 2006;24**:** 1187–92. 16685220 10.1097/01.hjh.0000226210.95936.bc

[25] Unal B Critchley JA Capewell S Modelling the decline in coronary heart disease deaths in England and Wales, 1981-2000: comparing contributions from primary prevention and secondary prevention. BMJ 2005;331**:** 614. 16107431 10.1136/bmj.38561.633345.8F PMC1215556

[26] Havlik RJ Hubert HB Fabsitz RR Feinleib M Weight and hypertension. Ann Intern Med 1983;98(5 Pt 2):855–59. 6847025 10.7326/0003-4819-98-5-855

[27] Wills AK Hardy RJ Black S Kuh DJ Trajectories of overweight and body mass index in adulthood and blood pressure at age 53: the 1946 British birth cohort study. J Hypertens 2010;28**:** 679–86. 20042875 10.1097/HJH.0b013e328335de7b

[28] Danon-Hersch N Chiolero A Shamlaye C Paccaud F Bovet P Decreasing association between body mass index and blood pressure over time. Epidemiology 2007;18**:** 493–500. 17525694 10.1097/EDE.0b013e318063eebf

[29] Tu YK Summers LK Burley V Trends in the association between blood pressure and obesity in a Taiwanese population between 1996 and 2006. J Hum Hypertens 2011;25**:** 88–97. 20336150 10.1038/jhh.2010.33

[30] Atherton K Fuller E Shepherd P Strachan DP Power C Loss and representativeness in a biomedical survey at age 45 years: 1958 British birth cohort. J Epidemiol Community Health 2008;62**:** 216–23. 18272736 10.1136/jech.2006.058966

[31] Wadsworth ME Mann SL Rodgers B Kuh DJ Hilder WS Yusuf EJ Loss and representativeness in a 43 year follow up of a national birth cohort. J Epidemiol Community Health 1992;46**:** 300–04. 1645091 10.1136/jech.46.3.300 PMC1059572

[32] Li L Dangour AD Power C Early life influences on adult leg and trunk length in the 1958 British birth cohort. Am J Hum Biol 2007;19**:** 836–43. 17696141 10.1002/ajhb.20649

[33] Barker DJ Fetal and infant origins of adult disease. BMJ 1990;301**:** 1111. 2252919 10.1136/bmj.301.6761.1111 PMC1664286

[34] Kuulasmaa K Tunstall-Pedoe H Dobson A Estimation of contribution of changes in classic risk factors to trends in coronary-event rates across the WHO MONICA Project populations. Lancet 2000;355**:** 675–87. 10703799 10.1016/s0140-6736(99)11180-2

[35] Dyer AR Stamler J Shekelle RB Pulse pressure-II. Factors associated with follow-up values in three Chicago epidemiologic studies. J Chronic Dis 1982;35**:** 275–82. 7061683 10.1016/0021-9681(82)90083-2

[36] Imamura H Tanaka K Hirae C Relationship of cigarette smoking to blood pressure and serum lipids and lipoproteins in men. Clin Exp Pharmacol Physiol 1996;23**:** 397–402. 8713678 10.1111/j.1440-1681.1996.tb02748.x

[37] Department of Health. Choosing Health: Making Healthy Choice Easier. London: Department of Health, 2004.

[38] Unal B Critchley JA Fidan D Capewell S Life-years gained from modern cardiological treatments and population risk factor changes in England and Wales, 1981-2000. Am J Public Health 2005;95**:** 103–08. 15623868 10.2105/AJPH.2003.029579 PMC1449860

[39] Hardoon SL Whincup PH Wannamethee SG Lennon LT Capewell S Morris RW Assessing the impact of medication use on trends in major coronary risk factors in older British men: a cohort study. Eur J Cardiovasc Prev Rehabil 2010;17**:** 502–08. 20386311 10.1097/HJR.0b013e3283378865 PMC3194092

[40] DeWilde SJ Carey IM Shah SM Trend in blood pressure in England: good treatment or good luck? J Epidemiol Community Health 2012;66(Suppl 1):A41.

