# Supplementary material for: Life-course body mass index trajectories and blood pressure in mid life in two British birth cohorts: stronger associations in the later-born generation
Source: Int J Epidemiol. 2015 Jun 13;44(3):1018–26. doi: 10.1093/ije/dyv106 (PMC4521132; doi:10.1093/ije/dyv106)
Supplement: Supplementary Data [file supp_44_3_1018__index.html]

Life-course body mass index trajectories and blood pressure in mid life in two British birth cohorts: stronger associations in the later-born generation — Supplementary Data 

# Life-course body mass index trajectories and blood pressure in mid life in two British birth cohorts: stronger associations in the later-born generation

## Supplementary Data

files

- Supplementary Data - pdf file
