# Supplementary material for: Life-course body mass index trajectories and blood pressure in mid life in two British birth cohorts: stronger associations in the later-born generation
Source: Int J Epidemiol. 2015 Jun 13;44(3):1018–26. doi: 10.1093/ije/dyv106 (PMC4521132; doi:10.1093/ije/dyv106)

Supplementary Information

Estimating the associations between characteristics of BMI trajectories and BP using joint multivariate response models

We applied a joint modelling approach to three response variables: repeated BMI measures (log10 transformed to correct for the right skewness) and SBP and DBP levels at 45y. For the 1958 (both sexes) and 1946 (males) cohorts, we used one knot (t_0) to allow for different "childhood'' and "adulthood'' BMI curves. For adult SBP and DBP, we specified only an individual-specific (random) intercept (β_{3j} and β_{4j}) to represent BP values for individual *j*. The joint model log *BMI* and adult SBP and DBP is written as

$$\log BMI_{ij} = \beta_{0j} + \beta_{1j} t_{ij} + \beta_{2j}(t_{ij} - t_0) I_{t_{ij} > t_0} + e_{ij} \qquad \text{(i)}$$
$$SBP_j = \beta_{3j}$$
$$DBP_j = \beta_{4j}$$

where t_{ij} is the exact age at measurement *i*. The indicator $I_{t_{ij} > t_0}$ represents two age ranges (=1 for $t_{ij} > t_0$ or =0 otherwise).

For $\log BMI_{ij}$ there are individual-specific intercept β_{0j}, childhood slope β_{1j} (for $t_{ij} \le t_0$), and adult slope $\beta_{1j} + \beta_{2j}$ (for $t_{ij} > t_0$). Model (i) was fitted by assuming that the random coefficients $\boldsymbol{\beta_j} = (\beta_{0j}, \beta_{1j}, \beta_{2j}, \beta_{3j}, \beta_{4j})^T$ are independently and identically distributed (*iid*) across *j* and follow a multivariate normal distribution, with mean $\beta = (\beta_0, \beta_1, \beta_2, \beta_3, \beta_4)^T$ and individual-level variance-covariance matrix (unstructured)

$$\Sigma = \begin{bmatrix} \sigma_0^2 & \sigma_{01} & \sigma_{02} & \sigma_{03} & \sigma_{04} \\ & \sigma_1^2 & \sigma_{12} & \sigma_{13} & \sigma_{14} \\ & & \sigma_2^2 & \sigma_{23} & \sigma_{24} \\ & & & \sigma_3^2 & \sigma_{34} \\ & & & & \sigma_4^2 \end{bmatrix}$$

The errors e_{ij} are assumed to be *iid* across *i* and *j* with $e_{ij} \sim N(0, \sigma_e^2)$, and independent of random effects $\boldsymbol{\beta_j}$. The three response variables are dependent on each other. The covariance terms

represent the degree to which the individual intercept and slopes for $\log BMI_{ij}$ (β_{0j}, β_{1j}, β_{2j}) co-vary within themselves and with intercepts (or values) of SBP (β_{3j}) and DBP (β_{4j}).

The individual BMI characteristics of interest include individual-specific $\log BMI_{ij}$ at age t ($\beta_{0j} + \beta_{1j}t + e_{ij}$ for $t \le t_0$; $\beta_{0j} - \beta_{2j}t_0 + (\beta_{1j} + \beta_{2j})t + e_{ij}$ for $t > t_0$) and slopes for $\log BMI_{ij}$ (β_{1j} for $t \le t_0$ and $\beta_{1j} + \beta_{2j}$ for $t > t_0$).

For simplicity, we write $I_{t_{ij}>t_0} = I_0$ and $t - t_0 = \Delta t_0$. The correlation between *SBP* and $\log BMI$ is a function of age t:

$$\frac{\sigma_{03}+\sigma_{13}t+\sigma_{23}\Delta t_0 I_0}{\sqrt{[\sigma_0^2+2\sigma_{01}t+\sigma_1^2t^2+\sigma_2^2\Delta t_0^2 I_0+2(\sigma_{02}+\sigma_{12}t)\Delta t_0 I_0+\sigma_e^2]\cdot\sigma_3^2}} \qquad (1)$$

The correlation between *SBP* and each slope (β_{1j} or $\beta_{1j} + \beta_{2j}$) is written as

$$\frac{\sigma_{13}+\sigma_{23}I_0}{\sqrt{[\sigma_1^2+(2\sigma_{12+}\sigma_2^2)\cdot I_0]\cdot\sigma_3^2}} \qquad (2)$$

The regression coefficient for *SBP* on $\log BMI$ is

$$\frac{\sigma_{03}+\sigma_{13}t+\sigma_{23}\Delta t_0 I_0}{\sigma_0^2+2\sigma_{01}t+\sigma_1^2t^2+\sigma_2^2\Delta t_0^2 I_0+2(\sigma_{02}+\sigma_{12}t)\Delta t_0 I_0+\sigma_e^2} \qquad (3)$$

The regression coefficient for *SBP* on each slope is

$$\frac{\sigma_{13}+\sigma_{23}\cdot I_0}{\sigma_1^2+(2\sigma_{12+}\sigma_2^2)\cdot I_0} \qquad (4)$$

For females in the 1946 cohort, the joint model which includes an additional knot (t_1) is written as

$$\log BMI_{ij} = \beta_{0j} + \beta_{1j}t_{ij} + \beta_{2j}(t_{ij} - t_0)I_{t_{ij}>t_0} + \beta_{2'j}(t_{ij} - t_1)I_{t_{ij}>t_1} + e_{ij} \qquad \text{(ii)}$$

$$SBP_j = \beta_{3j}$$

$$DBP_j = \beta_{4j} .$$

The indicator $I_{t_{ij}>t_1}$ takes the value 1 for $t_{ij}>t_1$ or 0 otherwise. In model (ii), the variance-covariance matrix for random coefficients $\boldsymbol{\beta}_j = (\beta_{0j}, \beta_{1j}, \beta_{2j}, \beta_{2'j}, \beta_{3j}, \beta_{4j})^T$ is

$$\Sigma = \begin{bmatrix} \sigma_0^2 & \sigma_{01} & \sigma_{02} & \sigma_{02'} & \sigma_{03} & \sigma_{04} \\ & \sigma_1^2 & \sigma_{12} & \sigma_{12'} & \sigma_{13} & \sigma_{14} \\ & & \sigma_2^2 & \sigma_{22'} & \sigma_{23} & \sigma_{24} \\ & & & \sigma_{2'}^2 & \sigma_{2'3} & \sigma_{2'4} \\ & & & & \sigma_3^2 & \sigma_{34} \\ & & & & & \sigma_4^2 \end{bmatrix}$$

The individual $\log BMI_{ij}$ is written as $\beta_{0j}+\beta_{1j}t+e_{ij}$ for $t\le t_0$, $\beta_{0j}-\beta_{2j}t_0+(\beta_{1j}+\beta_{2j})t+e_{ij}$ for $t_0<t<t_1$, or $\beta_{0j}-\beta_{2j}t_0-\beta_{2'j}t_1+(\beta_{1j}+\beta_{2j}+\beta_{2'j})t+e_{ij}$ for $t\ge t_1$, and three slopes as β_{1j}, $\beta_{1j}+\beta_{2j}$, and $\beta_{1j}+\beta_{2j}+\beta_{2'j}$ respectively.

Let $I_{t_{ij}>t_1}=I_1$ and $t-t_1=\Delta t_1$. The correlation between SBP and log *BMI* at age *t* is written as

$$\frac{\sigma_{03}+\sigma_{13}t+\sigma_{23}\Delta t_0 I_0+\sigma_{2'3}\Delta t_1 I_1}{\sqrt{[\sigma_0^2+2\sigma_{01}t+\sigma_1^2t^2+2(\sigma_{02}+\sigma_{12}t)\Delta t_0 I_0+\sigma_2^2\Delta t_0^2 I_0+2(\sigma_{02'}+\sigma_{12'}t+\sigma_{22'}\Delta t_0)\Delta t_1 I_1+\sigma_{2'}^2\Delta t_1^2 I_1+\sigma_e^2]\cdot\sigma_3^2}} \quad (5)$$

The correlation between SBP and each slope (β_{1j}, $\beta_{1j}+\beta_{2j}$, and $\beta_{1j}+\beta_{2j}+\beta_{2'j}$) can be written as

$$\frac{\sigma_{13}+\sigma_{23}I_0+\sigma_{2'3}I_1}{\sqrt{[\sigma_1^2+(2\sigma_{12}+\sigma_2^2)I_0+(2\sigma_{12'}+2\sigma_{22'}+\sigma_{2'}^2)I_1]\cdot\sigma_3^2}} \quad (6)$$

The regression coefficient for SBP on log *BMI* at age *t* is written as

$$\frac{\sigma_{03}+\sigma_{13}t+\sigma_{23}\Delta t_0 I_0+\sigma_{2'3}\Delta t_1 I_1}{\sigma_0^2+2\sigma_{01}t+\sigma_1^2t^2+\sigma_2^2\Delta t_0^2 I_0+2(\sigma_{02}+\sigma_{12}t)\Delta t_0 I_0+2(\sigma_{02'}+\sigma_{12'}t+\sigma_{22'}\Delta t_0)\Delta t_1 I_1+\sigma_{2'}^2\Delta t_1^2 I_1+\sigma_e^2} \quad (7)$$

The regression coefficient for SBP on each slope is

$$\frac{\sigma_{13}+\sigma_{23}I_0+\sigma_{2'3}I_1}{\sigma_1^2+(2\sigma_{12}+\sigma_2^2)I_0+(2\sigma_{12'}+2\sigma_{22'}+\sigma_{2'}^2)I_1} \quad (8)$$

Models (i) and (ii) were fitted in MLwiN. Model parameters were first estimated using the iterative generalized least squares algorithm which provides maximum likelihood estimates. The knot(s) was

positioned at different ages and -2 log(likelihood) of each model was compared. For model (i), the location of knot t_0 was chosen at 20y for males of both cohorts and at 16y for females of the 1958 cohort based on the likelihood profile (Figures S1a&S2). For model (ii), the knots t_0 and t_1 were chosen at 16 and 34y respectively (Figure S1b). Thus BMI trajectories for females of the 1946 cohort were characterised by three linear curves, with slopes β_{1j} (≤16y), $\beta_{1j}+\beta_{2j}$ (16y<*t*<34y) and $\beta_{1j}+\beta_{2j}+\beta_{2'j}$ (*t*≥34y). The residual plots for the random coefficients $\boldsymbol{\beta_j}$ in models (i) and (ii) suggest that it is reasonable to assume that they follow a normal distribution (Figures S2 and S3).

The correlations coefficients (or regression coefficients) estimated from Formulae 1-8 are non-linear functions of the model parameters. Their theoretical distributions are complex and the explicit formulae of variances cannot be expressed. We applied the nonparametric bootstrap re-sampling procedure. Each sample was drawn (with replacement) from estimated residuals at each level to create a dataset that has the same multilevel structure. These sampled residuals were then added to the fixed part of the model to obtain a new set of responses. Estimates of BMI levels and childhood and adult slopes and their correlations with BP were obtained for each of the bootstrap sample. We re-sampled 499 bootstrap datasets to obtain the means and 95% CI for the correlations coefficients. Similarly, the correlations between growth characteristics of BMI and DBP can be obtained by replacing the parameters for SBP in Models (i)&(ii) with those for DBP.

Table S1: Regression coefficients‡ for BP on BMI slopes estimated using joint multivariate models

	Systolic BP at 45y			
	1946 cohort		1958 cohort	
**Males**	Coefficient	95% CI	Coefficient	95% CI
Child slope (7-20y) †*	0.282	(-0.175,0.793)	1.039	(0.844,1.251)
Adult slope (>20y) †*	1.478	(0.717,2.269)	2.568	(2.148,3.097)
**Females**				
Child slope (7-16y) †	0.594	(0.092,1.166)	0.920	(0.545,1.321)
Adult slope (16-34y)†*	0.492	(-0.060,1.033)	2.274	(2.035,2.538)
Adult slope (>34y) †*	0.059	(-0.717,0.806)		
**Males**	**Diastolic BP at 45y**			
Child slope (7-20y) †	0.648	(0.245,1.027)	0.623	(0.534,0.722)
Adult slope (>20y) †	1.657	(1.046,2.205)	2.021	(1.756,2.371)
**Females**				
Child slope (7-16y) †	0.455	(0.055,0.877)	0.607	(0.364,0.851)
Adult slope (16-34y)†*	0.537	(0.130,0.939)	1.629	(1.469,1.791)
Adult slope (>34y) †*	0.069	(-0.484,0.582)		

† slope for log *BMI*
‡ change in BP for an increase of slope for log BMI by 0.001
* p<0.05 for test of the difference in regression coefficients between cohorts

Figure S1: Likelihood profile for the 1946 cohort for knots at different ages

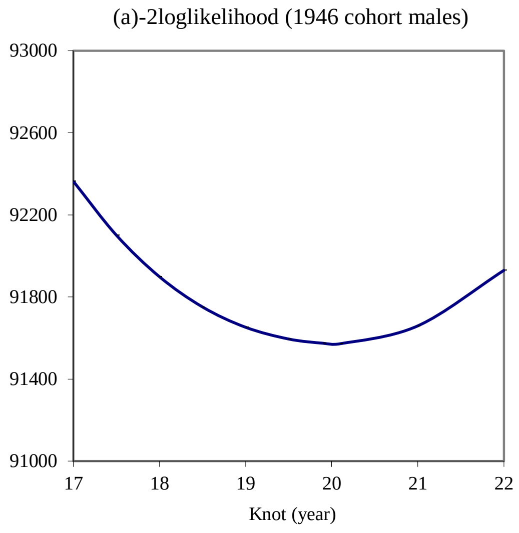

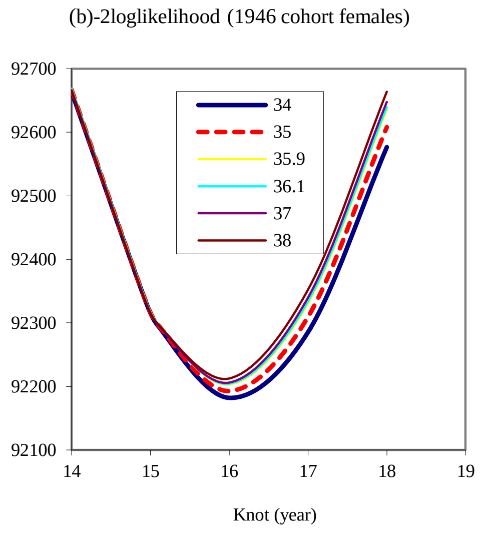

Figure S2: Likelihood profile for the 1958 cohort for knots at different ages

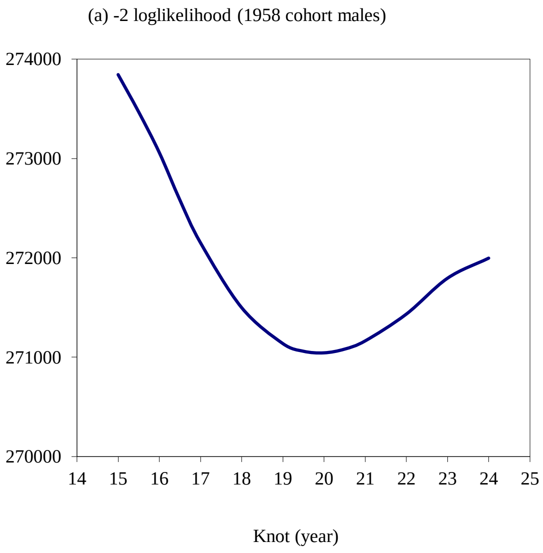

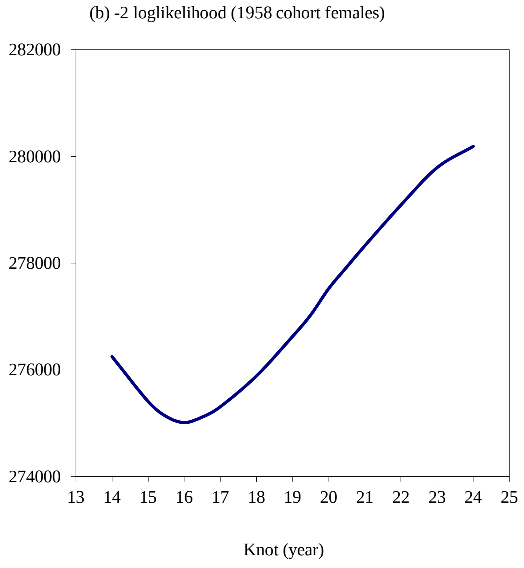

Figure S2: Residual plots for random coefficients in joint multivariate models for the 1946 cohort

Residual plots for $\beta_{0j}, \beta_{1j}, \beta_{2j}, \beta_{3j}, \beta_{4j}$ (males)

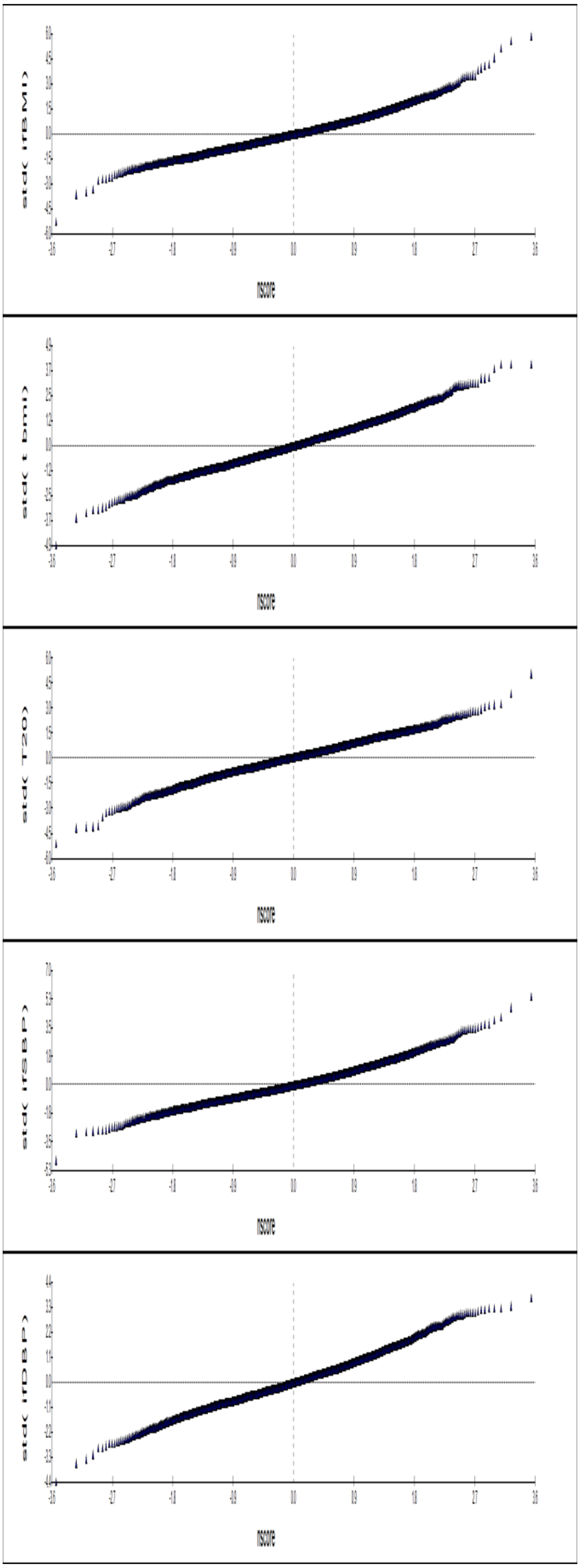

Residual plots for $\beta_{0j}, \beta_{1j}, \beta_{2j}, \beta_{2'j}, \beta_{3j}, \beta_{4j}$ (females)

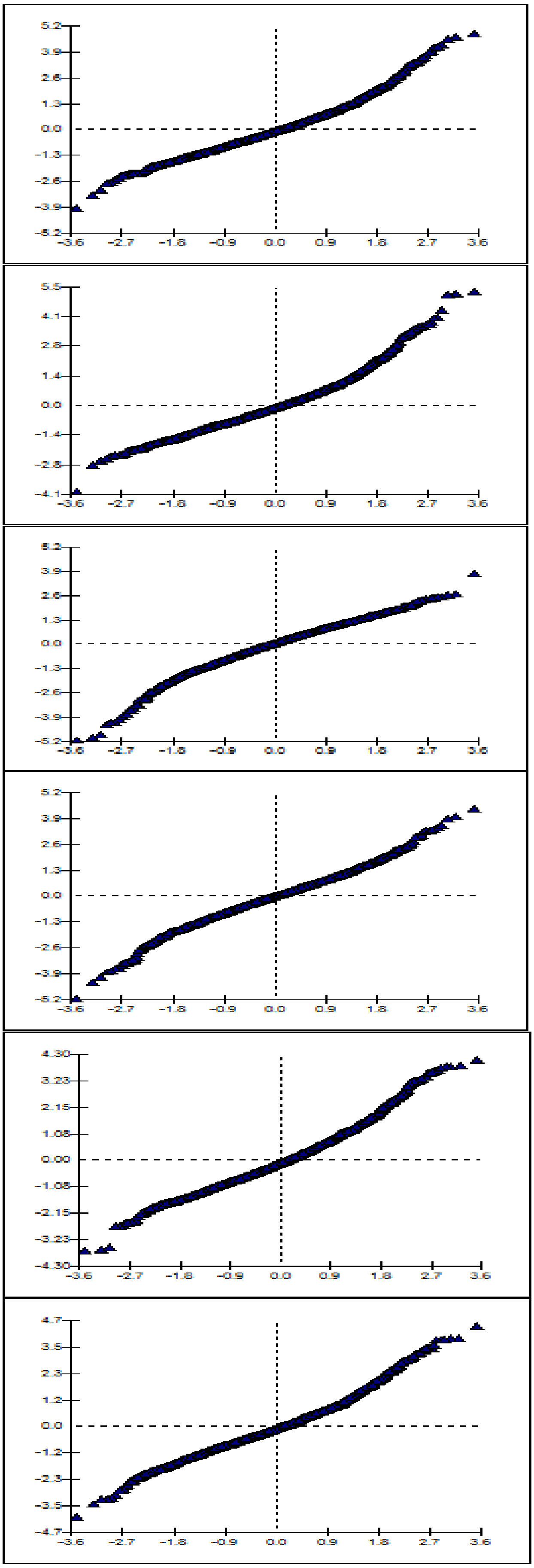

Figure S3: Residual plots for random coefficients in joint multivariate models for the 1958 cohort

Residual plots for $\beta_{0j}, \beta_{1j}, \beta_{2j}, \beta_{3j}, \beta_{4j}$ (males)

Residual plots for $\beta_{0j}, \beta_{1j}, \beta_{2j}, \beta_{3j}, \beta_{4j}$ (females)

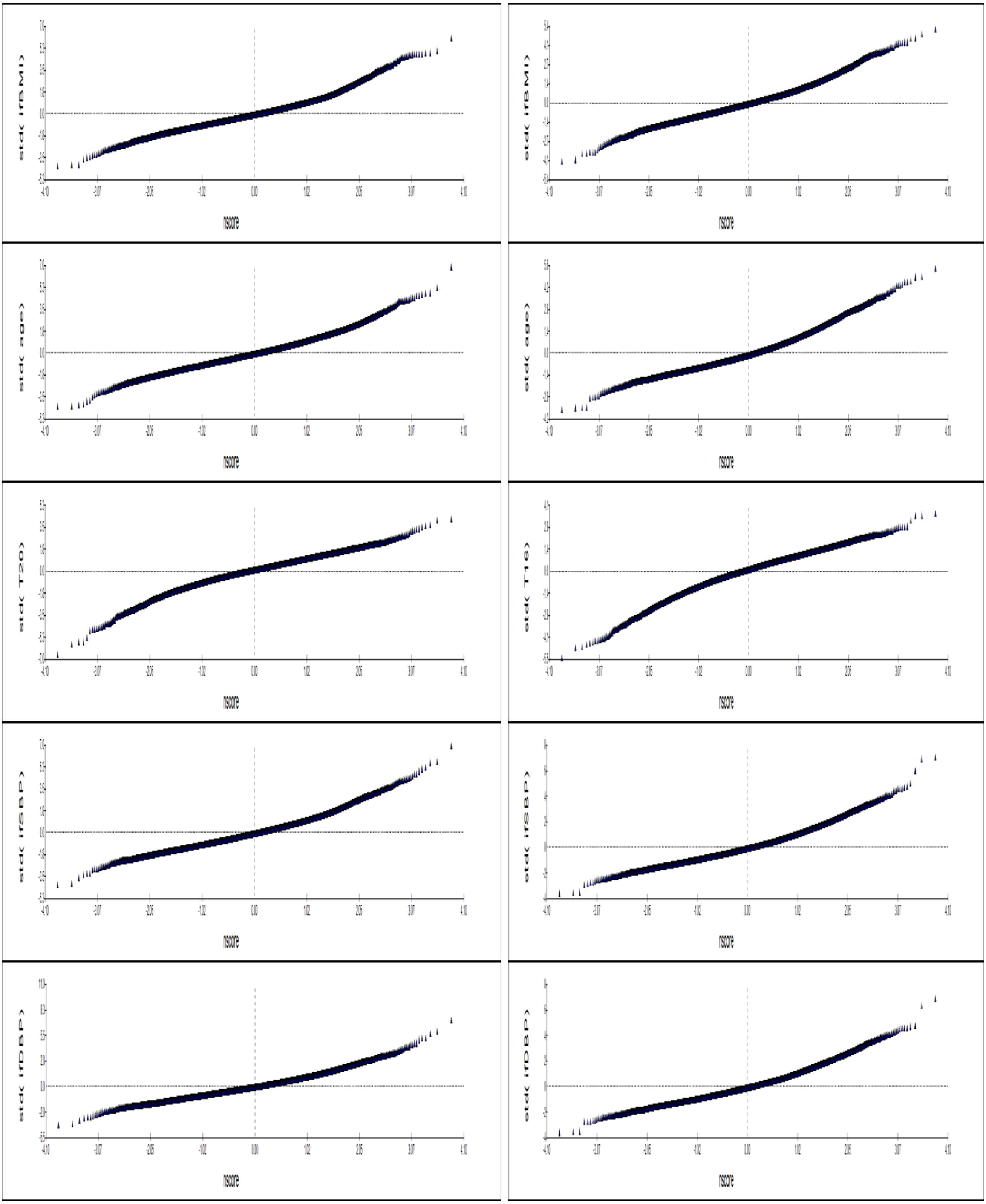

Supplement: Supplementary Data [file supp_dyv106_ije-2014-04-0365-File002.pdf]
